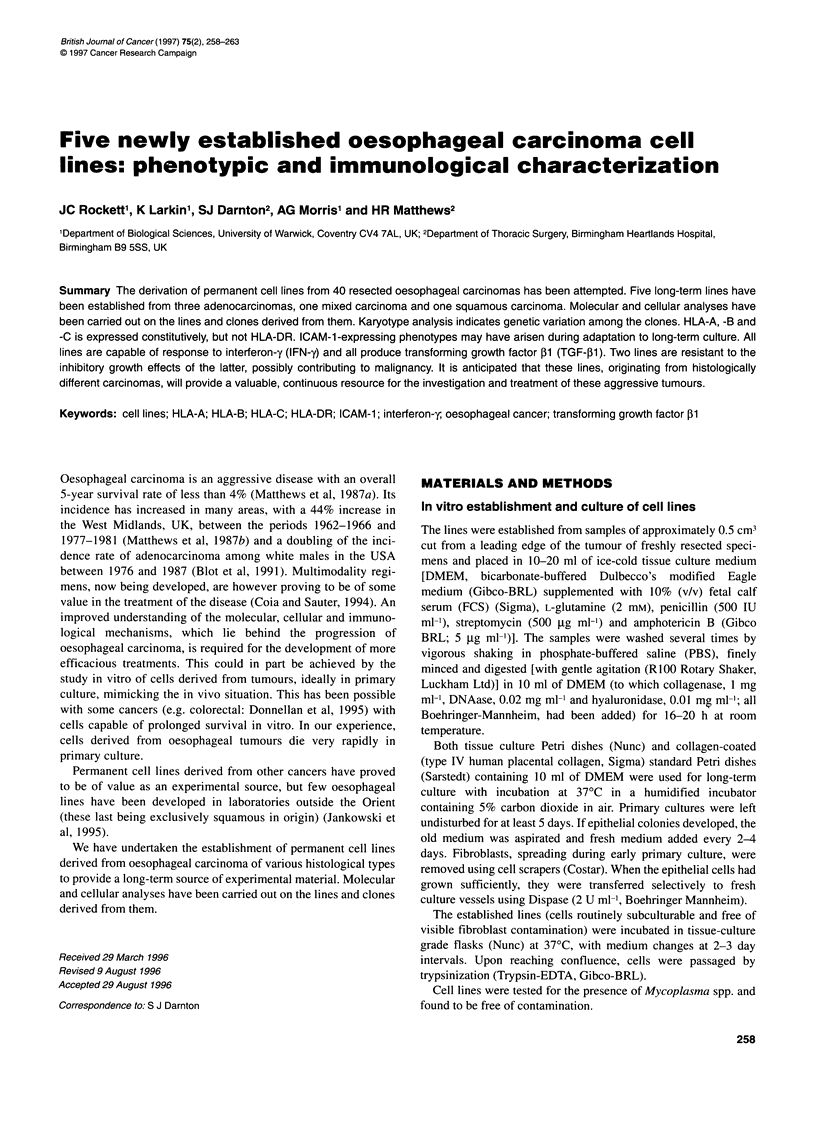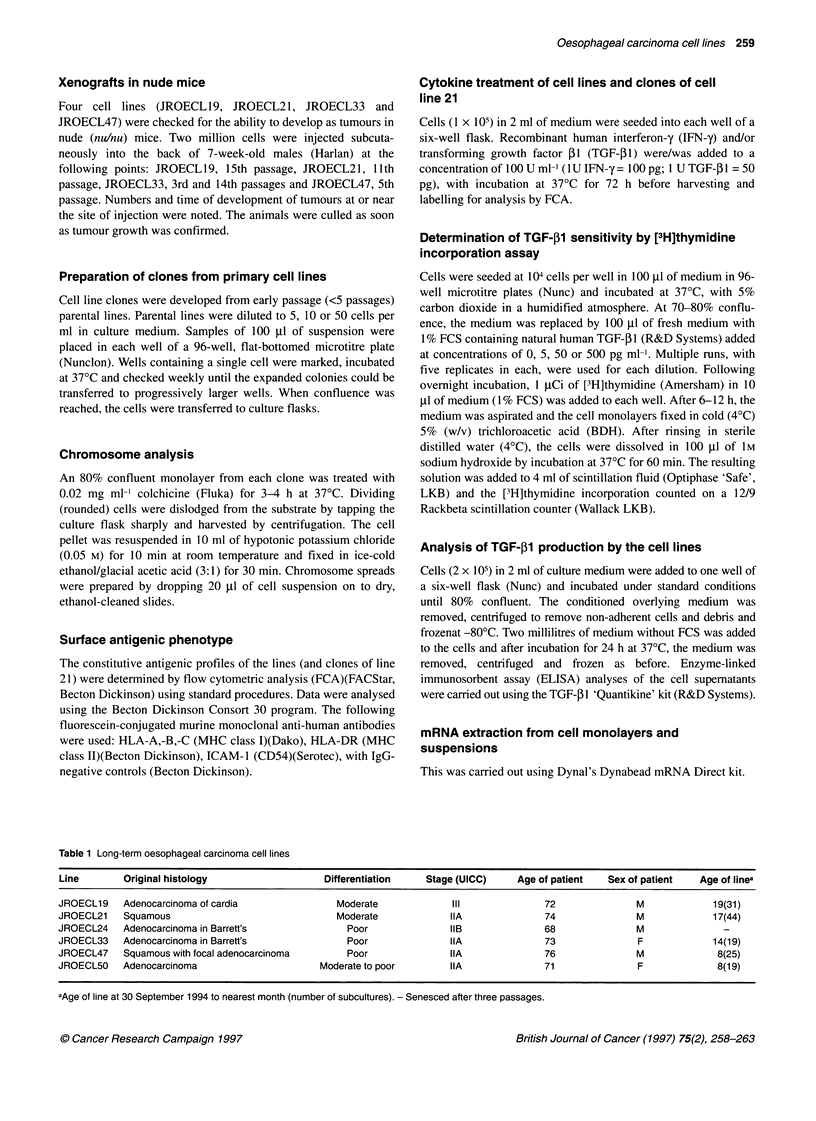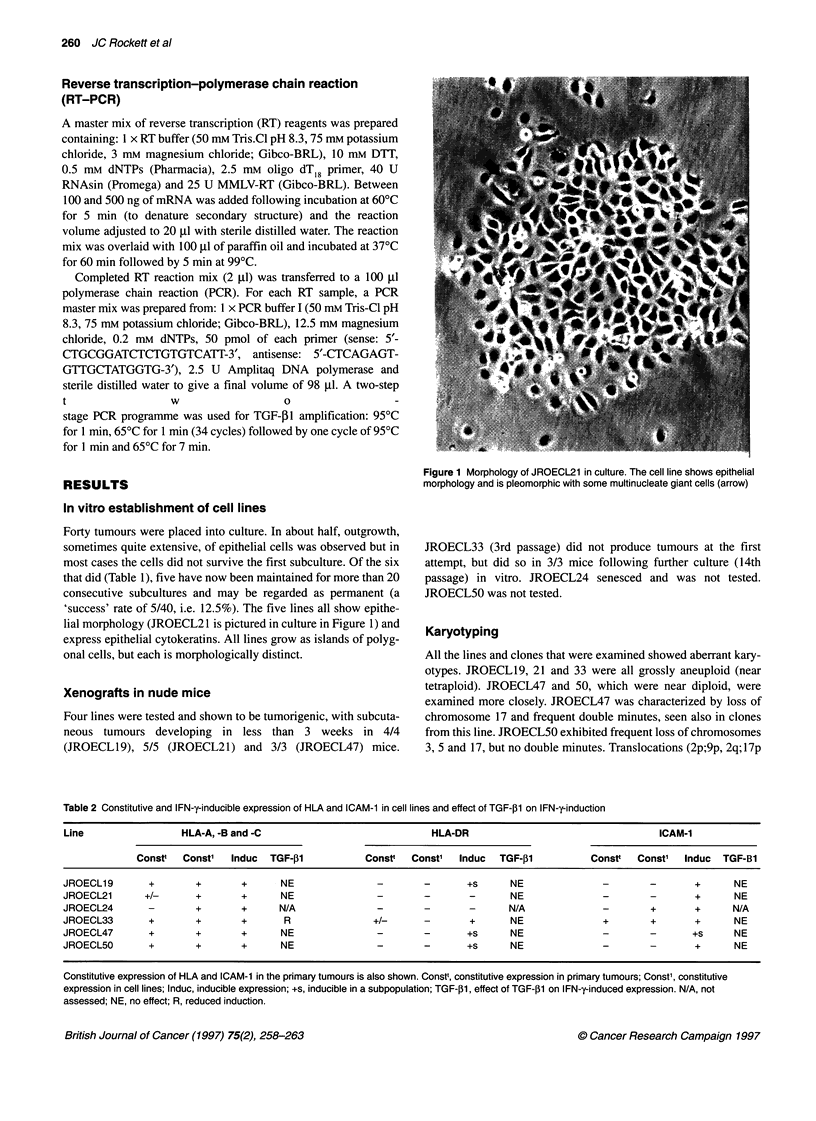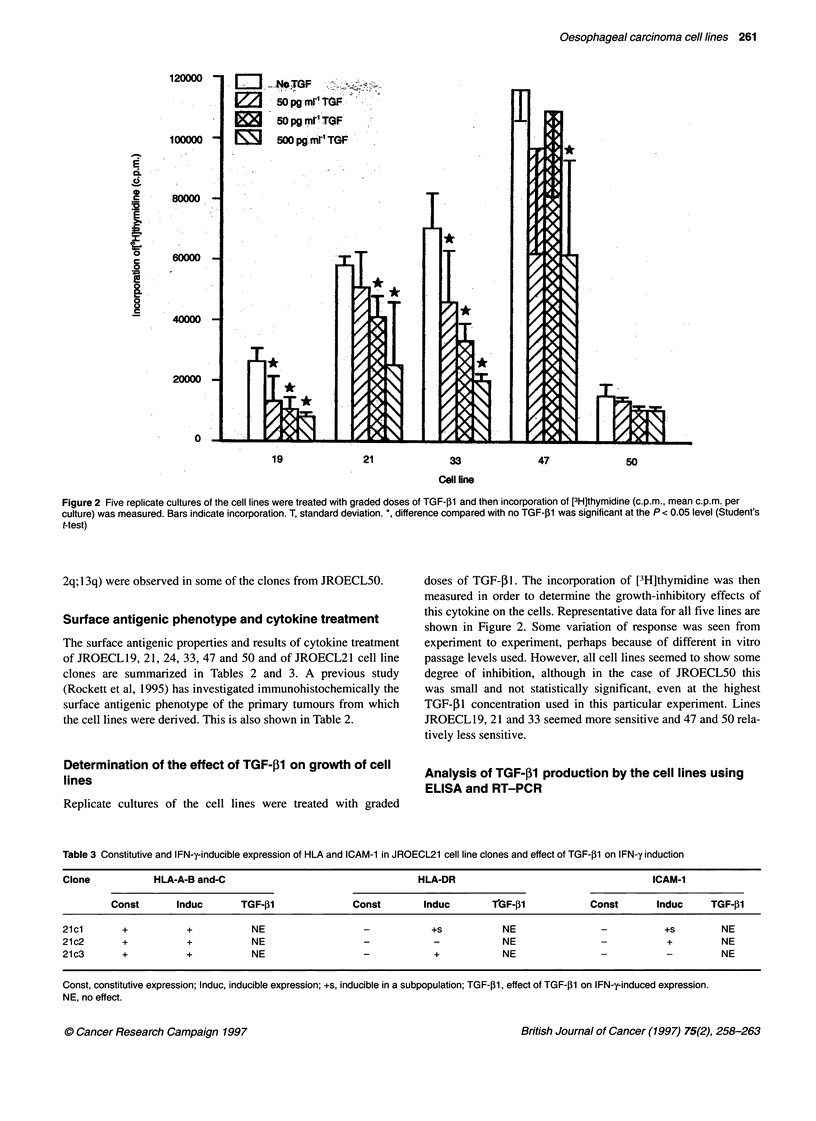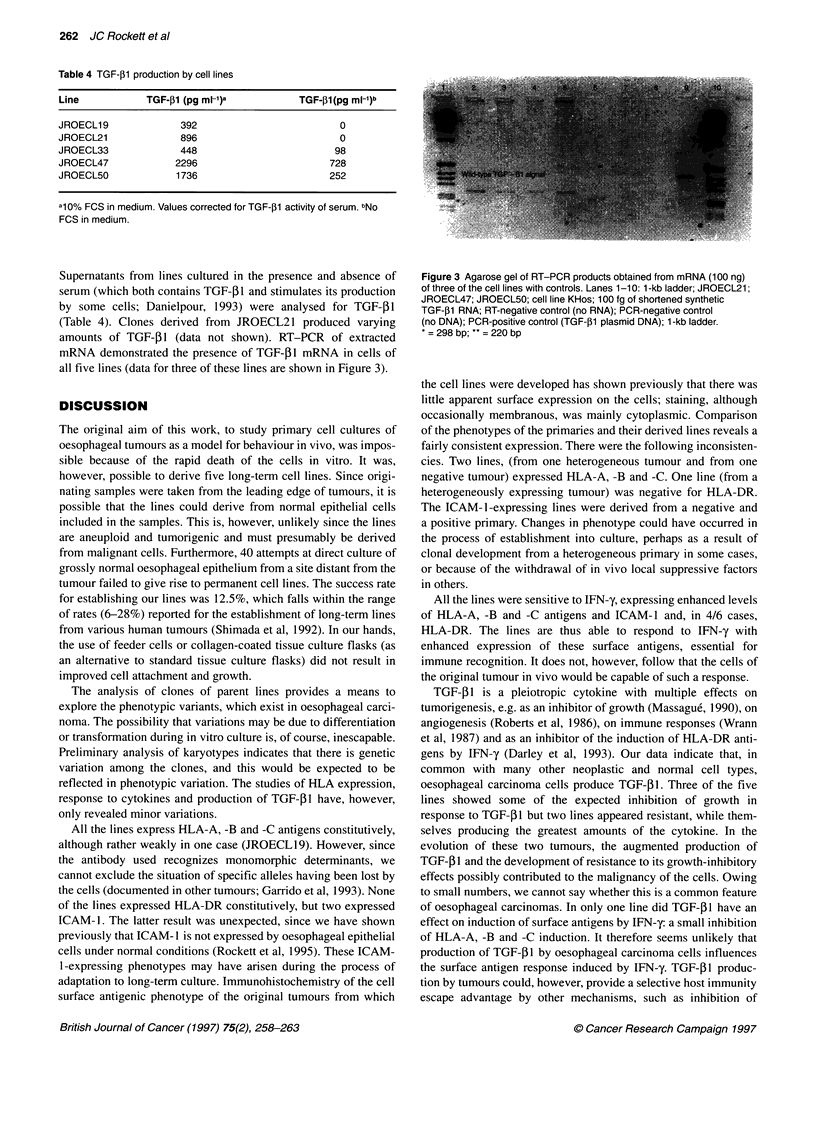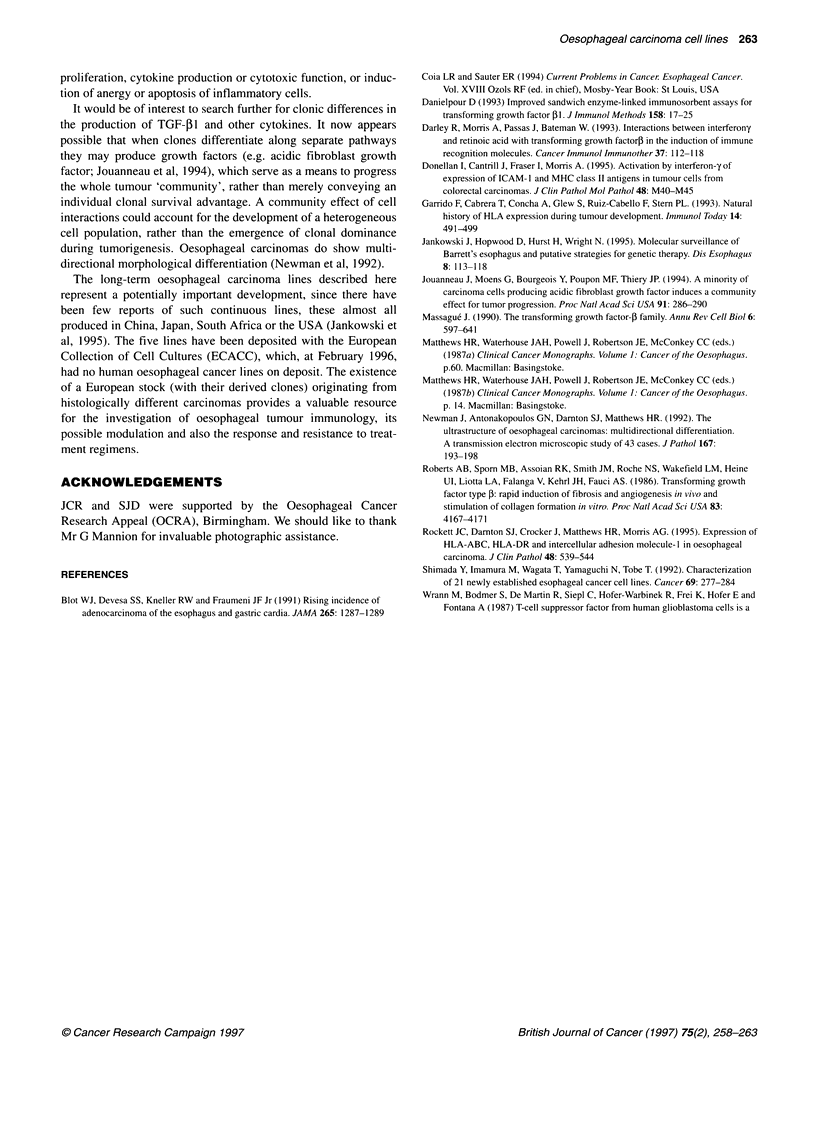# Five newly established oesophageal carcinoma cell lines: phenotypic and immunological characterization.

**DOI:** 10.1038/bjc.1997.42

**Published:** 1997

**Authors:** J. C. Rockett, K. Larkin, S. J. Darnton, A. G. Morris, H. R. Matthews

**Affiliations:** Department of Biological Sciences, University of Warwick, Coventry, UK.

## Abstract

**Images:**


British Journal of Cancer (1997) 75(2), 258–263
© 1997 Cancer Research Campaign

### Five newly established oesophageal carcinoma cell lines: phenotypic and immunological characterization

**JC Rockett[1], K Larkin[1], SJ Darnton[2], AG Morris[1] and HR Matthews[2]**

[1]Department of Biological Sciences, University of Warwick, Coventry CV4 7AL, UK; [2]Department of Thoracic Surgery, Birmingham Heartlands Hospital, Birmingham B9 5SS, UK

**Summary** The derivation of permanent cell lines from 40 resected oesophageal carcinomas has been attempted. Five long-term lines have been established from three adenocarcinomas, one mixed carcinoma and one squamous carcinoma. Molecular and cellular analyses have been carried out on the lines and clones derived from them. Karyotype analysis indicates genetic variation among the clones. HLA-A, -B and -C is expressed constitutively, but not HLA-DR. ICAM-1-expressing phenotypes may have arisen during adaptation to long-term culture. All lines are capable of response to interferon-γ (IFN-γ) and all produce transforming growth factor β1 (TGF-β1). Two lines are resistant to the inhibitory growth effects of the latter, possibly contributing to malignancy. It is anticipated that these lines, originating from histologically different carcinomas, will provide a valuable, continuous resource for the investigation and treatment of these aggressive tumours.

**Keywords:** cell lines; HLA-A; HLA-B; HLA-C; HLA-DR; ICAM-1; interferon-γ; oesophageal cancer; transforming growth factor β1

Oesophageal carcinoma is an aggressive disease with an overall 5-year survival rate of less than 4% (Matthews et al, 1987*a*). Its incidence has increased in many areas, with a 44% increase in the West Midlands, UK, between the periods 1962–1966 and 1977–1981 (Matthews et al, 1987*b*) and a doubling of the incidence rate of adenocarcinoma among white males in the USA between 1976 and 1987 (Blot et al, 1991). Multimodality regimens, now being developed, are however proving to be of some value in the treatment of the disease (Coia and Sauter, 1994). An improved understanding of the molecular, cellular and immunological mechanisms, which lie behind the progression of oesophageal carcinoma, is required for the development of more efficacious treatments. This could in part be achieved by the study in vitro of cells derived from tumours, ideally in primary culture, mimicking the in vivo situation. This has been possible with some cancers (e.g. colorectal: Donnellan et al, 1995) with cells capable of prolonged survival in vitro. In our experience, cells derived from oesophageal tumours die very rapidly in primary culture.

Permanent cell lines derived from other cancers have proved to be of value as an experimental source, but few oesophageal lines have been developed in laboratories outside the Orient (these last being exclusively squamous in origin) (Jankowski et al, 1995).

We have undertaken the establishment of permanent cell lines derived from oesophageal carcinoma of various histological types to provide a long-term source of experimental material. Molecular and cellular analyses have been carried out on the lines and clones derived from them.

*Received 29 March 1996*
*Revised 9 August 1996*
*Accepted 29 August 1996*

*Correspondence to:* S J Darnton

### MATERIALS AND METHODS

### In vitro establishment and culture of cell lines

The lines were established from samples of approximately 0.5 $cm^3$ cut from a leading edge of the tumour of freshly resected specimens and placed in 10–20 ml of ice-cold tissue culture medium [DMEM, bicarbonate-buffered Dulbecco's modified Eagle medium (Gibco-BRL) supplemented with 10% (v/v) fetal calf serum (FCS) (Sigma), L-glutamine (2 mM), penicillin (500 IU $ml^{-1}$), streptomycin (500 μg $ml^{-1}$) and amphotericin B (Gibco BRL; 5 μg $ml^{-1}$)]. The samples were washed several times by vigorous shaking in phosphate-buffered saline (PBS), finely minced and digested [with gentle agitation (R100 Rotary Shaker, Luckham Ltd)] in 10 ml of DMEM (to which collagenase, 1 mg $ml^{-1}$, DNAase, 0.02 mg $ml^{-1}$ and hyaluronidase, 0.01 mg $ml^{-1}$; all Boehringer-Mannheim, had been added) for 16–20 h at room temperature.

Both tissue culture Petri dishes (Nunc) and collagen-coated (type IV human placental collagen, Sigma) standard Petri dishes (Sarstedt) containing 10 ml of DMEM were used for long-term culture with incubation at 37°C in a humidified incubator containing 5% carbon dioxide in air. Primary cultures were left undisturbed for at least 5 days. If epithelial colonies developed, the old medium was aspirated and fresh medium added every 2–4 days. Fibroblasts, spreading during early primary culture, were removed using cell scrapers (Costar). When the epithelial cells had grown sufficiently, they were transferred selectively to fresh culture vessels using Dispase (2 U $ml^{-1}$, Boehringer Mannheim).

The established lines (cells routinely subculturable and free of visible fibroblast contamination) were incubated in tissue-culture grade flasks (Nunc) at 37°C, with medium changes at 2–3 day intervals. Upon reaching confluence, cells were passaged by trypsinization (Trypsin-EDTA, Gibco-BRL).

Cell lines were tested for the presence of *Mycoplasma* spp. and found to be free of contamination.

### Xenografts in nude mice

Four cell lines (JROECL19, JROECL21, JROECL33 and JROECL47) were checked for the ability to develop as tumours in nude (*nu/nu*) mice. Two million cells were injected subcutaneously into the back of 7-week-old males (Harlan) at the following points: JROECL19, 15th passage, JROECL21, 11th passage, JROECL33, 3rd and 14th passages and JROECL47, 5th passage. Numbers and time of development of tumours at or near the site of injection were noted. The animals were culled as soon as tumour growth was confirmed.

### Preparation of clones from primary cell lines

Cell line clones were developed from early passage (<5 passages) parental lines. Parental lines were diluted to 5, 10 or 50 cells per ml in culture medium. Samples of 100 µl of suspension were placed in each well of a 96-well, flat-bottomed microtitre plate (Nunclon). Wells containing a single cell were marked, incubated at 37°C and checked weekly until the expanded colonies could be transferred to progressively larger wells. When confluence was reached, the cells were transferred to culture flasks.

### Chromosome analysis

An 80% confluent monolayer from each clone was treated with 0.02 mg ml$^{-1}$ colchicine (Fluka) for 3–4 h at 37°C. Dividing (rounded) cells were dislodged from the substrate by tapping the culture flask sharply and harvested by centrifugation. The cell pellet was resuspended in 10 ml of hypotonic potassium chloride (0.05 M) for 10 min at room temperature and fixed in ice-cold ethanol/glacial acetic acid (3:1) for 30 min. Chromosome spreads were prepared by dropping 20 µl of cell suspension on to dry, ethanol-cleaned slides.

### Surface antigenic phenotype

The constitutive antigenic profiles of the lines (and clones of line 21) were determined by flow cytometric analysis (FCA)(FACStar, Becton Dickinson) using standard procedures. Data were analysed using the Becton Dickinson Consort 30 program. The following fluorescein-conjugated murine monoclonal anti-human antibodies were used: HLA-A,-B,-C (MHC class I)(Dako), HLA-DR (MHC class II)(Becton Dickinson), ICAM-1 (CD54)(Serotec), with IgG-negative controls (Becton Dickinson).

### Cytokine treatment of cell lines and clones of cell line 21

Cells ($1 \times 10^5$) in 2 ml of medium were seeded into each well of a six-well flask. Recombinant human interferon-γ (IFN-γ) and/or transforming growth factor β1 (TGF-β1) were/was added to a concentration of 100 U ml$^{-1}$ (1U IFN-γ = 100 pg; 1 U TGF-β1 = 50 pg), with incubation at 37°C for 72 h before harvesting and labelling for analysis by FCA.

### Determination of TGF-β1 sensitivity by [$^3$H]thymidine incorporation assay

Cells were seeded at $10^4$ cells per well in 100 µl of medium in 96-well microtitre plates (Nunc) and incubated at 37°C, with 5% carbon dioxide in a humidified atmosphere. At 70–80% confluence, the medium was replaced by 100 µl of fresh medium with 1% FCS containing natural human TGF-β1 (R&D Systems) added at concentrations of 0, 5, 50 or 500 pg ml$^{-1}$. Multiple runs, with five replicates in each, were used for each dilution. Following overnight incubation, 1 µCi of [$^3$H]thymidine (Amersham) in 10 µl of medium (1% FCS) was added to each well. After 6–12 h, the medium was aspirated and the cell monolayers fixed in cold (4°C) 5% (w/v) trichloroacetic acid (BDH). After rinsing in sterile distilled water (4°C), the cells were dissolved in 100 µl of 1M sodium hydroxide by incubation at 37°C for 60 min. The resulting solution was added to 4 ml of scintillation fluid (Optiphase 'Safe', LKB) and the [$^3$H]thymidine incorporation counted on a 12/9 Rackbeta scintillation counter (Wallack LKB).

### Analysis of TGF-β1 production by the cell lines

Cells ($2 \times 10^5$) in 2 ml of culture medium were added to one well of a six-well flask (Nunc) and incubated under standard conditions until 80% confluent. The conditioned overlying medium was removed, centrifuged to remove non-adherent cells and debris and frozenat –80°C. Two millilitres of medium without FCS was added to the cells and after incubation for 24 h at 37°C, the medium was removed, centrifuged and frozen as before. Enzyme-linked immunosorbent assay (ELISA) analyses of the cell supernatants were carried out using the TGF-β1 'Quantikine' kit (R&D Systems).

### mRNA extraction from cell monolayers and suspensions

This was carried out using Dynal's Dynabead mRNA Direct kit.

**Table 1** Long-term oesophageal carcinoma cell lines

| Line | Original histology | Differentiation | Stage (UICC) | Age of patient | Sex of patient | Age of line[a] |
|---|---|---|---|---|---|---|
| JROECL19 | Adenocarcinoma of cardia | Moderate | III | 72 | M | 19(31) |
| JROECL21 | Squamous | Moderate | IIA | 74 | M | 17(44) |
| JROECL24 | Adenocarcinoma in Barrett's | Poor | IIB | 68 | M | – |
| JROECL33 | Adenocarcinoma in Barrett's | Poor | IIA | 73 | F | 14(19) |
| JROECL47 | Squamous with focal adenocarcinoma | Poor | IIA | 76 | M | 8(25) |
| JROECL50 | Adenocarcinoma | Moderate to poor | IIA | 71 | F | 8(19) |

[a]Age of line at 30 September 1994 to nearest month (number of subcultures). – Senesced after three passages.

© Cancer Research Campaign 1997

### Reverse transcription–polymerase chain reaction (RT–PCR)

A master mix of reverse transcription (RT) reagents was prepared containing: 1 × RT buffer (50 mM Tris.Cl pH 8.3, 75 mM potassium chloride, 3 mM magnesium chloride; Gibco-BRL), 10 mM DTT, 0.5 mM dNTPs (Pharmacia), 2.5 mM oligo $dT_{18}$ primer, 40 U RNAsin (Promega) and 25 U MMLV-RT (Gibco-BRL). Between 100 and 500 ng of mRNA was added following incubation at 60°C for 5 min (to denature secondary structure) and the reaction volume adjusted to 20 µl with sterile distilled water. The reaction mix was overlaid with 100 µl of paraffin oil and incubated at 37°C for 60 min followed by 5 min at 99°C.

Completed RT reaction mix (2 µl) was transferred to a 100 µl polymerase chain reaction (PCR). For each RT sample, a PCR master mix was prepared from: 1 × PCR buffer I (50 mM Tris-Cl pH 8.3, 75 mM potassium chloride; Gibco-BRL), 12.5 mM magnesium chloride, 0.2 mM dNTPs, 50 pmol of each primer (sense: 5′-CTGCGGATCTCTGTGTCATT-3′, antisense: 5′-CTCAGAGT-GTTGCTATGGTG-3′), 2.5 U Amplitaq DNA polymerase and sterile distilled water to give a final volume of 98 µl. A two-step t w o - stage PCR programme was used for TGF-β1 amplification: 95°C for 1 min, 65°C for 1 min (34 cycles) followed by one cycle of 95°C for 1 min and 65°C for 7 min.

**Figure 1** Morphology of JROECL21 in culture. The cell line shows epithelial morphology and is pleomorphic with some multinucleate giant cells (arrow)

### RESULTS

### In vitro establishment of cell lines

Forty tumours were placed into culture. In about half, outgrowth, sometimes quite extensive, of epithelial cells was observed but in most cases the cells did not survive the first subculture. Of the six that did (Table 1), five have now been maintained for more than 20 consecutive subcultures and may be regarded as permanent (a 'success' rate of 5/40, i.e. 12.5%). The five lines all show epithelial morphology (JROECL21 is pictured in culture in Figure 1) and express epithelial cytokeratins. All lines grow as islands of polygonal cells, but each is morphologically distinct.

### Xenografts in nude mice

Four lines were tested and shown to be tumorigenic, with subcutaneous tumours developing in less than 3 weeks in 4/4 (JROECL19), 5/5 (JROECL21) and 3/3 (JROECL47) mice. JROECL33 (3rd passage) did not produce tumours at the first attempt, but did so in 3/3 mice following further culture (14th passage) in vitro. JROECL24 senesced and was not tested. JROECL50 was not tested.

### Karyotyping

All the lines and clones that were examined showed aberrant karyotypes. JROECL19, 21 and 33 were all grossly aneuploid (near tetraploid). JROECL47 and 50, which were near diploid, were examined more closely. JROECL47 was characterized by loss of chromosome 17 and frequent double minutes, seen also in clones from this line. JROECL50 exhibited frequent loss of chromosomes 3, 5 and 17, but no double minutes. Translocations (2p;9p, 2q;17p

**Table 2** Constitutive and IFN-γ-inducible expression of HLA and ICAM-1 in cell lines and effect of TGF-β1 on IFN-γ-induction

| Line | HLA-A, -B and -C | | | | HLA-DR | | | | ICAM-1 | | | |
|---|---|---|---|---|---|---|---|---|---|---|---|---|
| | Const$^t$ | Const$^1$ | Induc | TGF-β1 | Const$^t$ | Const$^1$ | Induc | TGF-β1 | Const$^t$ | Const$^1$ | Induc | TGF-B1 |
| JROECL19 | + | + | + | NE | – | – | +s | NE | – | – | + | NE |
| JROECL21 | +/– | + | + | NE | – | – | – | NE | – | – | + | NE |
| JROECL24 | – | + | + | N/A | – | – | – | N/A | – | + | + | N/A |
| JROECL33 | + | + | + | R | +/– | – | + | NE | + | + | + | NE |
| JROECL47 | + | + | + | NE | – | – | +s | NE | – | – | +s | NE |
| JROECL50 | + | + | + | NE | – | – | +s | NE | – | – | + | NE |

Constitutive expression of HLA and ICAM-1 in the primary tumours is also shown. Const$^t$, constitutive expression in primary tumours; Const$^1$, constitutive expression in cell lines; Induc, inducible expression; +s, inducible in a subpopulation; TGF-β1, effect of TGF-β1 on IFN-γ-induced expression. N/A, not assessed; NE, no effect; R, reduced induction.

 © Cancer Research Campaign 1997

**Figure 2** Five replicate cultures of the cell lines were treated with graded doses of TGF-β1 and then incorporation of [$^3$H]thymidine (c.p.m., mean c.p.m. per culture) was measured. Bars indicate incorporation. T, standard deviation. *, difference compared with no TGF-β1 was significant at the $P < 0.05$ level (Student's *t*-test)

2q;13q) were observed in some of the clones from JROECL50.

### Surface antigenic phenotype and cytokine treatment

The surface antigenic properties and results of cytokine treatment of JROECL19, 21, 24, 33, 47 and 50 and of JROECL21 cell line clones are summarized in Tables 2 and 3. A previous study (Rockett et al, 1995) has investigated immunohistochemically the surface antigenic phenotype of the primary tumours from which the cell lines were derived. This is also shown in Table 2.

### Determination of the effect of TGF-β1 on growth of cell lines

Replicate cultures of the cell lines were treated with graded doses of TGF-β1. The incorporation of [$^3$H]thymidine was then measured in order to determine the growth-inhibitory effects of this cytokine on the cells. Representative data for all five lines are shown in Figure 2. Some variation of response was seen from experiment to experiment, perhaps because of different in vitro passage levels used. However, all cell lines seemed to show some degree of inhibition, although in the case of JROECL50 this was small and not statistically significant, even at the highest TGF-β1 concentration used in this particular experiment. Lines JROECL19, 21 and 33 seemed more sensitive and 47 and 50 relatively less sensitive.

### Analysis of TGF-β1 production by the cell lines using ELISA and RT–PCR

**Table 3** Constitutive and IFN-γ-inducible expression of HLA and ICAM-1 in JROECL21 cell line clones and effect of TGF-β1 on IFN-γ induction

| Clone | HLA-A-B and-C | | | HLA-DR | | | ICAM-1 | | |
|---|---|---|---|---|---|---|---|---|---|
| | Const | Induc | TGF-β1 | Const | Induc | TGF-β1 | Const | Induc | TGF-β1 |
| 21c1 | + | + | NE | – | +s | NE | – | +s | NE |
| 21c2 | + | + | NE | – | – | NE | – | + | NE |
| 21c3 | + | + | NE | – | + | NE | – | – | NE |

Const, constitutive expression; Induc, inducible expression; +s, inducible in a subpopulation; TGF-β1, effect of TGF-β1 on IFN-γ-induced expression. NE, no effect.

© Cancer Research Campaign 1997 

**Table 4** TGF-β1 production by cell lines

| Line | TGF-β1 (pg ml$^{-1}$)[a] | TGF-β1(pg ml$^{-1}$)[b] |
|---|---|---|
| JROECL19 | 392 | 0 |
| JROECL21 | 896 | 0 |
| JROECL33 | 448 | 98 |
| JROECL47 | 2296 | 728 |
| JROECL50 | 1736 | 252 |

[a]10% FCS in medium. Values corrected for TGF-β1 activity of serum. [b]No FCS in medium.

**Figure 3** Agarose gel of RT–PCR products obtained from mRNA (100 ng) of three of the cell lines with controls. Lanes 1–10: 1-kb ladder; JROECL21; JROECL47; JROECL50; cell line KHos; 100 fg of shortened synthetic TGF-β1 RNA; RT-negative control (no RNA); PCR-negative control (no DNA); PCR-positive control (TGF-β1 plasmid DNA); 1-kb ladder. * = 298 bp; ** = 220 bp

Supernatants from lines cultured in the presence and absence of serum (which both contains TGF-β1 and stimulates its production by some cells; Danielpour, 1993) were analysed for TGF-β1 (Table 4). Clones derived from JROECL21 produced varying amounts of TGF-β1 (data not shown). RT–PCR of extracted mRNA demonstrated the presence of TGF-β1 mRNA in cells of all five lines (data for three of these lines are shown in Figure 3).

### DISCUSSION

The original aim of this work, to study primary cell cultures of oesophageal tumours as a model for behaviour in vivo, was impossible because of the rapid death of the cells in vitro. It was, however, possible to derive five long-term cell lines. Since originating samples were taken from the leading edge of tumours, it is possible that the lines could derive from normal epithelial cells included in the samples. This is, however, unlikely since the lines are aneuploid and tumorigenic and must presumably be derived from malignant cells. Furthermore, 40 attempts at direct culture of grossly normal oesophageal epithelium from a site distant from the tumour failed to give rise to permanent cell lines. The success rate for establishing our lines was 12.5%, which falls within the range of rates (6–28%) reported for the establishment of long-term lines from various human tumours (Shimada et al, 1992). In our hands, the use of feeder cells or collagen-coated tissue culture flasks (as an alternative to standard tissue culture flasks) did not result in improved cell attachment and growth.

The analysis of clones of parent lines provides a means to explore the phenotypic variants, which exist in oesophageal carcinoma. The possibility that variations may be due to differentiation or transformation during in vitro culture is, of course, inescapable. Preliminary analysis of karyotypes indicates that there is genetic variation among the clones, and this would be expected to be reflected in phenotypic variation. The studies of HLA expression, response to cytokines and production of TGF-β1 have, however, only revealed minor variations.

All the lines express HLA-A, -B and -C antigens constitutively, although rather weakly in one case (JROECL19). However, since the antibody used recognizes monomorphic determinants, we cannot exclude the situation of specific alleles having been lost by the cells (documented in other tumours; Garrido et al, 1993). None of the lines expressed HLA-DR constitutively, but two expressed ICAM-1. The latter result was unexpected, since we have shown previously that ICAM-1 is not expressed by oesophageal epithelial cells under normal conditions (Rockett et al, 1995). These ICAM-1-expressing phenotypes may have arisen during the process of adaptation to long-term culture. Immunohistochemistry of the cell surface antigenic phenotype of the original tumours from which the cell lines were developed has shown previously that there was little apparent surface expression on the cells; staining, although occasionally membranous, was mainly cytoplasmic. Comparison of the phenotypes of the primaries and their derived lines reveals a fairly consistent expression. There were the following inconsistencies. Two lines, (from one heterogeneous tumour and from one negative tumour) expressed HLA-A, -B and -C. One line (from a heterogeneously expressing tumour) was negative for HLA-DR. The ICAM-1-expressing lines were derived from a negative and a positive primary. Changes in phenotype could have occurred in the process of establishment into culture, perhaps as a result of clonal development from a heterogeneous primary in some cases, or because of the withdrawal of in vivo local suppressive factors in others.

All the lines were sensitive to IFN-γ, expressing enhanced levels of HLA-A, -B and -C antigens and ICAM-1 and, in 4/6 cases, HLA-DR. The lines are thus able to respond to IFN-γ with enhanced expression of these surface antigens, essential for immune recognition. It does not, however, follow that the cells of the original tumour in vivo would be capable of such a response.

TGF-β1 is a pleiotropic cytokine with multiple effects on tumorigenesis, e.g. as an inhibitor of growth (Massagué, 1990), on angiogenesis (Roberts et al, 1986), on immune responses (Wrann et al, 1987) and as an inhibitor of the induction of HLA-DR antigens by IFN-γ (Darley et al, 1993). Our data indicate that, in common with many other neoplastic and normal cell types, oesophageal carcinoma cells produce TGF-β1. Three of the five lines showed some of the expected inhibition of growth in response to TGF-β1 but two lines appeared resistant, while themselves producing the greatest amounts of the cytokine. In the evolution of these two tumours, the augmented production of TGF-β1 and the development of resistance to its growth-inhibitory effects possibly contributed to the malignancy of the cells. Owing to small numbers, we cannot say whether this is a common feature of oesophageal carcinomas. In only one line did TGF-β1 have an effect on induction of surface antigens by IFN-γ: a small inhibition of HLA-A, -B and -C induction. It therefore seems unlikely that production of TGF-β1 by oesophageal carcinoma cells influences the surface antigen response induced by IFN-γ. TGF-β1 production by tumours could, however, provide a selective host immunity escape advantage by other mechanisms, such as inhibition of

© Cancer Research Campaign 1997

proliferation, cytokine production or cytotoxic function, or induction of anergy or apoptosis of inflammatory cells.

It would be of interest to search further for clonic differences in the production of TGF-β1 and other cytokines. It now appears possible that when clones differentiate along separate pathways they may produce growth factors (e.g. acidic fibroblast growth factor; Jouanneau et al, 1994), which serve as a means to progress the whole tumour 'community', rather than merely conveying an individual clonal survival advantage. A community effect of cell interactions could account for the development of a heterogeneous cell population, rather than the emergence of clonal dominance during tumorigenesis. Oesophageal carcinomas do show multidirectional morphological differentiation (Newman et al, 1992).

The long-term oesophageal carcinoma lines described here represent a potentially important development, since there have been few reports of such continuous lines, these almost all produced in China, Japan, South Africa or the USA (Jankowski et al, 1995). The five lines have been deposited with the European Collection of Cell Cultures (ECACC), which, at February 1996, had no human oesophageal cancer lines on deposit. The existence of a European stock (with their derived clones) originating from histologically different carcinomas provides a valuable resource for the investigation of oesophageal tumour immunology, its possible modulation and also the response and resistance to treatment regimens.

### ACKNOWLEDGEMENTS

JCR and SJD were supported by the Oesophageal Cancer Research Appeal (OCRA), Birmingham. We should like to thank Mr G Mannion for invaluable photographic assistance.

### REFERENCES

Blot WJ, Devesa SS, Kneller RW and Fraumeni JF Jr (1991) Rising incidence of adenocarcinoma of the esophagus and gastric cardia. *JAMA* **265**: 1287–1289

Coia LR and Sauter ER (1994) *Current Problems in Cancer. Esophageal Cancer.* Vol. XVIII Ozols RF (ed. in chief), Mosby-Year Book: St Louis, USA

Danielpour D (1993) Improved sandwich enzyme-linked immunosorbent assays for transforming growth factor β1. *J Immunol Methods* **158**: 17–25

Darley R, Morris A, Passas J, Bateman W. (1993). Interactions between interferonγ and retinoic acid with transforming growth factorβ in the induction of immune recognition molecules. *Cancer Immunol Immunother* **37**: 112–118

Donellan I, Cantrill J, Fraser I, Morris A. (1995). Activation by interferon-γ of expression of ICAM-1 and MHC class II antigens in tumour cells from colorectal carcinomas. *J Clin Pathol Mol Pathol* **48**: M40–M45

Garrido F, Cabrera T, Concha A, Glew S, Ruiz-Cabello F, Stern PL. (1993). Natural history of HLA expression during tumour development. *Immunol Today* **14**: 491–499

Jankowski J, Hopwood D, Hurst H, Wright N. (1995). Molecular surveillance of Barrett's esophagus and putative strategies for genetic therapy. *Dis Esophagus* **8**: 113–118

Jouanneau J, Moens G, Bourgeois Y, Poupon MF, Thiery JP. (1994). A minority of carcinoma cells producing acidic fibroblast growth factor induces a community effect for tumor progression. *Proc Natl Acad Sci USA* **91**: 286–290

Massagué J. (1990). The transforming growth factor-β family. *Annu Rev Cell Biol* **6**: 597–641

Matthews HR, Waterhouse JAH, Powell J, Robertson JE, McConkey CC (eds.) (1987*a*) *Clinical Cancer Monographs. Volume 1: Cancer of the Oesophagus.* p.60. Macmillan: Basingstoke.

Matthews HR, Waterhouse JAH, Powell J, Robertson JE, McConkey CC (eds.) (1987*b*) *Clinical Cancer Monographs. Volume 1: Cancer of the Oesophagus.* p. 14. Macmillan: Basingstoke.

Newman J, Antonakopoulos GN, Darnton SJ, Matthews HR. (1992). The ultrastructure of oesophageal carcinomas: multidirectional differentiation. A transmission electron microscopic study of 43 cases. *J Pathol* **167**: 193–198

Roberts AB, Sporn MB, Assoian RK, Smith JM, Roche NS, Wakefield LM, Heine UI, Liotta LA, Falanga V, Kehrl JH, Fauci AS. (1986). Transforming growth factor type β: rapid induction of fibrosis and angiogenesis *in vivo* and stimulation of collagen formation *in vitro*. *Proc Natl Acad Sci USA* **83**: 4167–4171

Rockett JC, Darnton SJ, Crocker J, Matthews HR, Morris AG. (1995). Expression of HLA-ABC, HLA-DR and intercellular adhesion molecule-1 in oesophageal carcinoma. *J Clin Pathol* **48**: 539–544

Shimada Y, Imamura M, Wagata T, Yamaguchi N, Tobe T. (1992). Characterization of 21 newly established esophageal cancer cell lines. *Cancer* **69**: 277–284

Wrann M, Bodmer S, De Martin R, Siepl C, Hofer-Warbinek R, Frei K, Hofer E and Fontana A (1987) T-cell suppressor factor from human glioblastoma cells is a

© Cancer Research Campaign 1997